# Pretreatment with Roxadustat (FG-4592) Attenuates Folic Acid-Induced Kidney Injury through Antiferroptosis via Akt/GSK-3*β*/Nrf2 Pathway

**DOI:** 10.1155/2020/6286984

**Published:** 2020-01-17

**Authors:** Xue Li, Yu Zou, Jia Xing, Yuan-Yuan Fu, Kai-Yue Wang, Peng-Zhi Wan, Xiao-Yue Zhai

**Affiliations:** ^1^Department of Histology and Embryology, Basic Medical College, China Medical University, Shenyang, Liaoning, China; ^2^Department of Nephrology, Shengjing Hospital of China Medical University, Shenyang, Liaoning, China; ^3^Department of Nephrology, First Affiliated Hospital of China Medical University, Shenyang, Liaoning, China; ^4^Institute of Nephropathology, China Medical University, Shenyang, China

## Abstract

Folic acid- (FA-) induced kidney injury is characterized by the tubule damage due to the disturbance of the antioxidant system and subsequent interstitial fibrosis. FG-4592 is an inhibitor of prolyl hydroxylase of hypoxia-inducible factor (HIF), an antioxidant factor. The present study investigated the protective role of FG-4592 pretreatment at the early stage of the kidney injury and long-term impact on the progression of renal fibrosis. FG-4592 was administrated two days before FA injection in mice. On the second day after FA injection, the mice with FG-4592 pretreatment showed an improved renal function, compared with those without FG-4592 pretreatment, indicated by biochemical and histological parameters; meanwhile, the cellular content of iron, malondialdehyde, and 4-hydroxynonenal histologically decreased, implying the suppression of iron accumulation and lipid peroxidation. Simultaneously, upregulation of HIF-1*α* was found, along with Nrf2 activation, which was reflected by increased nuclear translocation and high-expression of downstream proteins, including heme-oxygenase1, glutathione peroxidase4, and cystine/glutamate transporter, as well as ferroportin. Correspondingly, the elevated levels of antioxidative enzymes and glutathione, as well as reduced iron accumulation, were observed, suggesting a lower risk of occurrence of ferroptosis with FG-4592 pretreatment. This was confirmed by reversed pathological parameters and improved renal function in FA-treated mice with the administration of ferrostatin-1, a specific ferroptosis inhibitor. Furthermore, a signal pathway study indicated that Nrf2 activation was associated with increased phosphorylation of Akt and GSK-3*β*, verified by the use of an inhibitor of the PI3K that phosphorylates Akt. Moreover, FG-4592 pretreatment also decreased macrophage infiltration and expression of inflammatory factors TNF-*α* and IL-1*β*. On the 14^th^ day after FA injection, FG-4592 pretreatment decreased collagen deposition and expression of fibrosis biomarkers. These findings suggest that the protective role of FG-4592 pretreatment is achieved mainly by decreasing ferroptosis at the early stage of FA-induced kidney injury via Akt/GSK-3*β*-mediated Nrf2 activation, which retards the fibrosis progression.

## 1. Introduction

Acute kidney injury (AKI) is characterized by an acute and transient renal dysfunction and often progressively develops into chronic kidney disease (CKD) without adequate treatment [1–4]. At present, clinical approaches are insufficient to prevent the development of AKI [5]. FA-induced AKI is a widely used model for studying the mechanisms underlying nephrotoxic tubule damage and gradual progression of renal fibrosis [6, 7]. In the clinic, the use of high-dose folinic acid combining with other antitumor drugs for chemotherapy of metastatic gastrointestinal cancer has raised the incidence rate of FA-induced kidney injury [8]. Kidney injury caused by high-dose FA injection is mainly due to the direct toxicity to the epithelium and partly to the formation of luminal crystals. Water and vacuole degeneration and cellular swelling are the initial pathological features of tubular damage [9, 10]. These pathological features in mice are comparable with those in human kidney injury, and the experimental model recapitulates all the major processes in human kidney injury [11]. The production of reactive oxygen species (ROS) and consequent disturbance of the antioxidation system followed by apoptosis, ferroptosis, or even necrosis are the generally accepted mechanisms for oxidative stress-mediated kidney injury [12, 13]. Ferroptosis, which can be inhibited by specific inhibitors such as ferrostatin-1 (Fer-1), is characterized by iron-dependent lipid peroxidation [14]. Glutathione (GSH) and antioxidant enzymes can eliminate ROS and therefore decrease the level of lipid peroxidation, reflected mainly by two indexes, malondialdehyde (MDA) and 4-hydroxynonenal (4-HNE) [15]. In addition, the inflammatory responses caused by ferroptosis have been thought to be a driver for the occurrence of other cell death, such as apoptosis or necroptosis [16].

Nuclear factor erythroid 2-related factor 2 (Nrf2) plays an important role in antiferroptosis; it is a master regulator that transcriptionally regulates almost all Nrf2-antioxidant reactive element (ARE) pathways [17]. Also, Nrf2 regulates an iron export protein, ferroportin that modulates cellular iron homeostasis [18]. Activation of Nrf2 could upregulate a battery of ROS-detoxifying enzymes, such as heme-oxygenase1 (HO-1), glutathione peroxidase4 (GPX4), and quinone oxidoreductase (NQO1) etc. [19]. Of these, GPX4 participates in decreasing ferroptosis by promoting reduction of GSSG into GSH [20]. Moreover, cysteine/glutamate transporter, SLC7A11 that involves in GSH synthesis, is regulated by Nrf2 [21]. Nrf2 hyperactivation in the early phase of renal injury prevents tubular damage progression [22]. Kelch-like ECH-associated protein 1 (Keap1) is an inhibitory protein that binds to Nrf2 and promotes its degradation by the ubiquitin proteasome pathway which is the primary point of regulation in the Nrf2 pathway [23]. In addition to Keap1-dependent Nrf2 regulation, Keap1-independent regulatory pathways also play a key role in governing impaired Nrf2 activity. What is more, burgeoning evidence suggests that GSK-3*β* mediated Keap1-independent regulatory pathway is a key pathway involved in Nrf2 activation, thus protecting from kidney injury [24].

A hypoxia-inducible factor (HIF) is an endogenous antioxidative stress modulator that consists of a constitutively expressed *β* subunit and a short-lived, oxygen-regulated *α* subunit [25]. HIF can be degraded by prolyl hydroxylases (PHD) in normoxia [26]. HIF-1*α* precondition has been shown to enhance the antioxidant activity in neuroprotection [27]. Moreover, it has been reported that HIF-1*α* can activate the Nrf2-ARE pathway to protect from ischemia-reperfusion cardiac and skeletal muscle injuries [25, 28]. We therefore proposed that pharmacological preconditioning, aiming at activating and stabilizing endogenous HIF-1*α*, enhances antioxidant capacity and efficiently attenuates AKI.

FG-4592 is a novel, small-molecule stabilizer of HIF by inhibiting PHD that can hydroxylate the *α* subunit of HIF for degradation in normoxia [26]. Presently, FG-4592 is orally administered to CKD patients to improve the anemia [29]. In the present study, the protective role of FG-4592 pretreatment at the early stage of FA-induced kidney injury was demonstrated to be associated with HIF-1*α* stabilization and Nrf2 activation, thus retarding the progression of renal fibrosis. The underlying mechanisms were further investigated.

## 2. Materials and Methods

### 2.1. Animals

All animal experiments were conducted per the NIH Guidelines for the Care and Use of Laboratory Animals, approved by the local Institutional Animal Care and Use Committee. C57BL/6 male mice, 6 to 8 weeks old, were purchased from Liaoning Changsheng Biotechnology Co. (Liaoning, China). The animals were housed in controlled temperature and humidity according to a 12 h light/dark cycle. The animal experiment was conducted in three parts. In the first part, mice were randomly divided into 4 groups (*n* = 12/group): (1) control group that received an intraperitoneal injection of saline, (2) FG-4592 group that received intraperitoneal injection of FG-4592 once (10 mg/kg, dissolved in DMSO at 50 mg/ml and then further diluted in sterile phosphate-buffered saline to 1 mg/ml), (3) FA group that received intraperitoneal injection of a single dose of FA (250 mg/kg, dissolved in 0.3 M sodium bicarbonate), and (4) FA+FG-4592 group that received FG-4592 two days prior to FA single-dose injection. Kidney specimens and blood samples were collected on the second day (*n* = 6/group) and the fourteenth day (*n* = 6/group) after FA injection for further examination.

In the second part, mice were treated with a ferroptosis inhibitor (Fer-1). Mice were randomly divided into 3 groups (*n* = 6/group): (1) control group, (2) FA group, and (3) FA+Fer-1 group that received an intraperitoneal injection of Fer-1 (5 mg/kg) 30 minutes before FA injection. Kidney specimens and blood samples were collected on the second day (*n* = 6/group) after FA injection for further examination.

In the third part, mice were treated with a PI3K inhibitor (wortmannin). Mice were randomly divided into 4 groups (*n* = 6/group): (1) FA group, (2) FA+FG-4592 group, (3) FA+Wort group that received intraperitoneal injection of wortmannin (0.5 mg/kg) and FA, and (4) FA+FG-4592+Wort group that received FG-4592 two days prior to injection of wortmannin (0.5 mg/kg) and FA. Kidney specimens and blood samples were collected on the second day (*n* = 6/group) after FA injection for further examination.

### 2.2. Reagents and Antibodies

FG-4592 was purchased from Selleck (Houston, Texas, USA), while wortmannin and antibodies to p-Akt, Akt, p-GSK-3*β*, GSK-3*β*, IL-1*β*, and F4/80 were purchased from CST (Danvers, MA, USA). FA was obtained from Dalian Meilun Biotechnology Co. (Dalian, China), and Fer-1 was obtained from Santa Cruz Biotechnology (Santa Cruz, CA, USA). Antibodies to *β*-actin, HIF-1*α*, KIM-1, Nrf2, GPX4, 4-HNE, vimentin, fibronectin (Fn), SLC7A11, ferroportin, histone H3, and assay kits for Perls' iron staining were acquired from Abcam (Cambridge, MA, USA). Antibody to MDA was purchased from Novus Biologicals (Littleton, CO, USA). Antibodies to TNF-*α*, HO-1, and collagen IV were obtained from Proteintech (Wuhan, China). Antibody to Keap1 was acquired from Wanlei (Shenyang, China). Assay kits for GSH (A-006-2) and MDA (BC0025) were acquired from Solarbio (Beijing, China). Assay kits for creatinine (C011-2), urea nitrogen (BUN) (C013-2), and iron (A039-2) were obtained from Jiancheng (Nanjing, China). TUNEL assay was acquired from Roche (Basel, Switzerland). An assay kit for nuclear extract was purchased from Active Motif (Tokyo, Japan).

### 2.3. Assays for Renal Function and for Lipid Peroxidation, GSH, and Iron

Serum was prepared for blood BUN and creatinine measurement in accordance with the manufacturer's protocol to analyze renal function. Renal tissues were prepared for the quantitation of MDA, GSH, and iron using assay kits to assess the lipid peroxidation and iron homeostasis.

### 2.4. TUNEL Staining for Detecting Cell Death

Terminal deoxynucleotidyl transferase-mediated digoxigenin-deoxyuridine nick-end labeling (TUNEL) staining was performed in 3 *μ*m thick sections of paraffin-embedded tissue with the in situ cell death detection kit fluorescein according to the manufacturer's instructions.

### 2.5. Renal Tissue Preparation for Histopathologic Examination

The kidneys were fixed with paraformaldehyde overnight, dehydrated in graded ethanol, embedded in paraffin, and cut into 3 *μ*m thick sections. The sections were stained with hematoxylin and eosin (H&E) and Masson's trichrome (Jiancheng) to assess the histopathologic changes in the kidneys. The H&E sections were blindly examined to score signs of tubular damage, such as loss of brush border, tubular dilation, interstitial edema, cellular necrosis, and vacuolization. The criteria were as follows: 0, no damage; 1, <20%; 2, 20–50%; 3, 50–70%; and 4, >70%. For each animal, at least 10 fields were examined (Brooks, Wei, Cho, & Dong, 2009).

### 2.6. Immunohistochemical Staining for 4-HNE, MDA, GPX4, HO-1, F4/80, TNF-*α*, and IL-1*β*

The paraffin-embedded tissue sections were dewaxed in xylene and rehydrated in an ethanol gradient. Subsequently, sections were immersed in 0.01 M sodium citrate buffer (pH 6.0) or EDTA (pH = 8.0) and were boiled for 2 min 30 s at high power using a pressure cooker. The sections then were cooled at room temperature and washed in phosphate-buffered saline (PBS) 3 times for 5 min each time, followed by incubation with 3% H_2_O_2_ 10 min and goat serum for approximately 30 min preincubation. The sections were then incubated with primary antibodies, including anti-4-HNE (1 : 200), anti-MDA (1 : 50), anti-GPX4 (1 : 200), anti-HO-1(1 : 200), anti-TNF-*α* (1 : 100), anti-IL–1*β* (1 : 100), anti-F4/80 (1 : 250), anti-Fn (1 : 150), and anti-collagen IV (1 : 200) overnight at 4°C. On the next day, the sections were washed and incubated with biotinylated goat anti-rabbit/mouse IgG for 1 h. The reaction results were visualized with diaminobenzidine (DAB) (1809270031, MXB-BIO, Fuzhou, China). Hematoxylin was used as a counterstain. Images were taken with a camera mounted on a Nikon microscope (90i, Nikon, Tokyo, Japan).

### 2.7. Perls' Staining

Cellular iron accumulation was detected by Perls' staining, as described in a book (American Registry of Pathology, Prophet, Edna B, 1992). The image of iron accumulation was obtained using a Nikon microscope (90i, Nikon, Tokyo, Japan).

### 2.8. Immunofluorescence Staining for Nrf2

After deparaffinization, the sections were boiled in 0.01 M sodium citrate buffer (pH 6.0), then cooled at room temperature and washed in PBS 3 times for 5 min each. The tissue was then disintegrated for 10 min with 0.3% Triton-X-100 and then blocked in goat serum for approximately 30 min and subsequently incubated with the primary rabbit anti-Nrf2 antibody (1 : 150) overnight at 4°C. On the next day, the sections were washed and then incubated in the dark with TRITC-conjugated goat anti-rabbit IgG (1 : 200) (Dako, Glostrup, Denmark). Subsequently, the sections were exposed to DAPI for 5 min and then observed under a fluorescence microscope (Nikon 90i, Tokyo, Japan).

### 2.9. Western Blotting Analysis

A nuclear extract kit was used to separate nuclear and cytoplasmic proteins of kidney tissues, according to the manufacturer's protocol. Kidney general proteins were extracted with lysis buffer plus RIPA (Beyotime, Shanghai, China), 1% phenylmethylsulfonyl fluoride (Beyotime, China), and 1% phosphatase inhibitors (Solarbio). The protein concentration was determined using a BCA kit (Beyotime). Subsequently, 50 *μ*g of protein samples was separated by electrophoresis through a 10% sodium dodecyl sulfate-polyacrylamide gel. The samples were then transferred to a polyvinylidene difluoride membrane, which was blocked in 5% milk for 1 h at room temperature. The membrane was then incubated with primary antibodies, including anti-KIM-1 (1 : 500), anti-HIF-1*α* (1 : 500), anti-Nrf2 (1 : 1000), anti-4-HNE (1 : 3000), anti-GPX4 (1 : 5000), anti-HO-1 (1 : 1000), anti-Keap1 (1 : 500), anti-p-Akt (1 : 1000), anti-Akt (1 : 1000), anti-p-GSK3*β* (1 : 1000), anti-GSK3*β* (1 : 1000), anti-IL-1*β* (1 : 1000), anti-TNF-*α* (1 : 1000), anti-vimentin (1 : 500), anti-collagen IV (1 : 1000), anti-Fn (1 : 1000), anti-*β*-actin (1 : 3000), and anti-histone H3 (1 : 500) antibodies overnight at 4°C. The samples were incubated with horseradish peroxidase- (HRP-) conjugated goat anti-rabbit/anti-mouse secondary antibodies (1 : 10000, Dako) for 1 h at room temperature. The signals were detected via an enhanced chemiluminescence (ECL) system using an ECL kit. The expression levels were normalized to that of *β*-actin or histone H3 and were quantified with the Image-Pro Plus 6.0 program (Media Cybernetics, Bethesda, MD, USA).

### 2.10. Real-Time PCR Quantification for GPX4 and HO-1 mRNA

The kidney tissue (60 mg) was cut into pieces in 1 ml TRIzol solution (Vazyme, Nanjing, China) and then incubated with 0.2 ml of chloroform on ice for 5 min. The supernatants were extracted via centrifugation at 12,000 rpm for 15 min and were mixed with the same volume of isopropanol on ice for 15 min. The mixtures were centrifuged at 12,000 g for 10 min, and the upper phase was discarded. Next, 1 ml of 75% ethanol/ml TRIzol was added, and the solutions were centrifuged at 8,000 g for 5 min. DEPC water was added to the dissolved RNA. The RNA concentrations were determined and standardized to 1000 ng/*μ*l. The total RNA was reverse-transcribed (RT) to cDNA using a PrimeScript RT reagent kit (Vazyme). Subsequently, a polymerase chain reaction (PCR) was performed on the resulting cDNA with a SYBR Green Mix (Vazyme) on a Roche 4800 RT-PCR detection system. The specific primers (Dingguo Changsheng, Beijing, China) used were as follows: GPX4 forward: 5′-GGTTTCGTGTGCATCGTCAC-3′ and reverse: 5′-GGGCATCGTCCCCATTTACA-3′; HO-1 forward: 5′-AGGGCAGAAGGGAATTGCTC-3′ and reverse: 5′-AAAGAGCTGGAGAGCCAACC-3′; *β*-actin forward: 5′-GGCTGTATTCCCCTCCATCG-3′ and reverse: 5′-CCAGTTGGTAATGCCATGT-3′. The relative mRNA expression levels were determined using the 2^−*ΔΔ*Ct^ method.

### 2.11. Statistical Analysis

The data were shown as the means ± standard deviations (SD). All analyses were carried out using SPSS software, version 21.0 (SPSS Inc., Chicago, IL, USA). One-way analysis of variance followed by the Bonferroni test was used to compare the treated groups with the control group. A *P* value of <0.05 was considered statistically significant.

## 3. Results

### 3.1. FG-4592 Pretreatment Alleviated FA-Induced Tubular Damage and Renal Function

**Figure 1:**
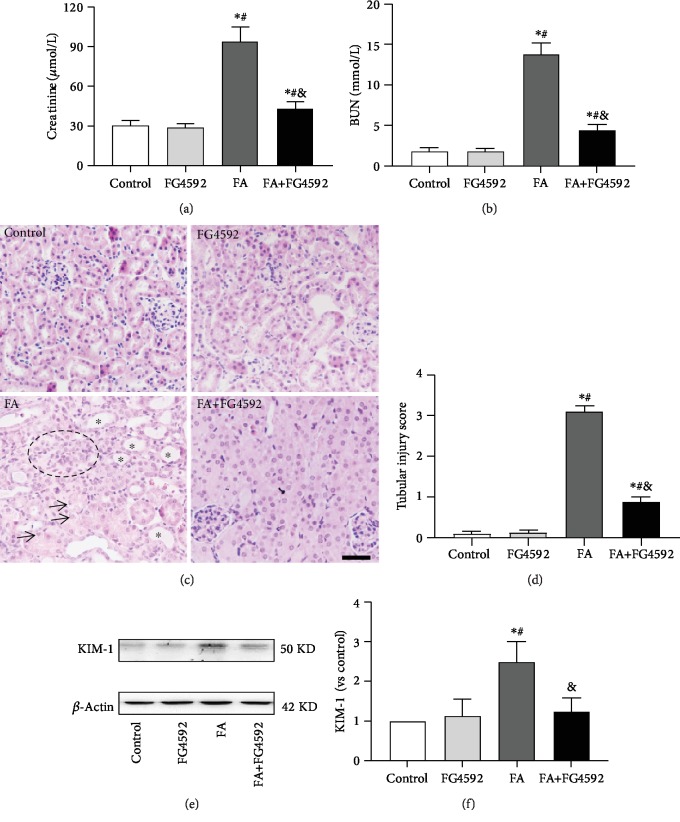
The renoprotective effects of FG-4592 pretreatment in FA-induced acute tubular damage. (a and b) Renal function is assessed by (a) plasma creatinine and (b) BUN levels. (c) Renal tubular injury was assessed by H&E staining. Bar, 50 *μ*m. Asterisks for dilated tubule; arrows for vacuole degenerated cells; black circle for inflammatory infiltration. (d) Kidney tubular injury score based on H&E staining. (e) Western blotting analysis of kidney injury molecule 1 (KIM-1). (f) Semiquantitative measurements of KIM-1. The values are expressed as the means ± SEM (*n* = 6). ^∗^*P* < 0.05 versus the control group. ^#^*P* < 0.05 versus the FG-4592 group. ^&^*P* < 0.05 versus the FA group.

Our present study indicated that the FA group showed increased serum levels of BUN, creatinine, and KIM-1, a marker of tubular damage, as compared to the control group on the second day after FA administration. Consistent with the results, the histological analysis of the FA group showed severe tubular damage. Some tubules were dilated with thin-walled epithelia and hyaline casts in their lumen, some tubule lumens were narrowed with swollen epithelial cells filled with multivacuoles, and exfoliative cells were seen in the lumen occasionally. In addition, interstitial infiltration of inflammatory cells was often observed around cortical vessels. The tubular damage was significantly improved in the FA+FG-4592 group. The results are summarized in Figure 1. Both the control group and the FG-4592 group showed normal kidney tissue morphology.

### 3.2. FG-4592 Pretreatment Decreased Ferroptosis in FA-Induced AKI

**Figure 2:**
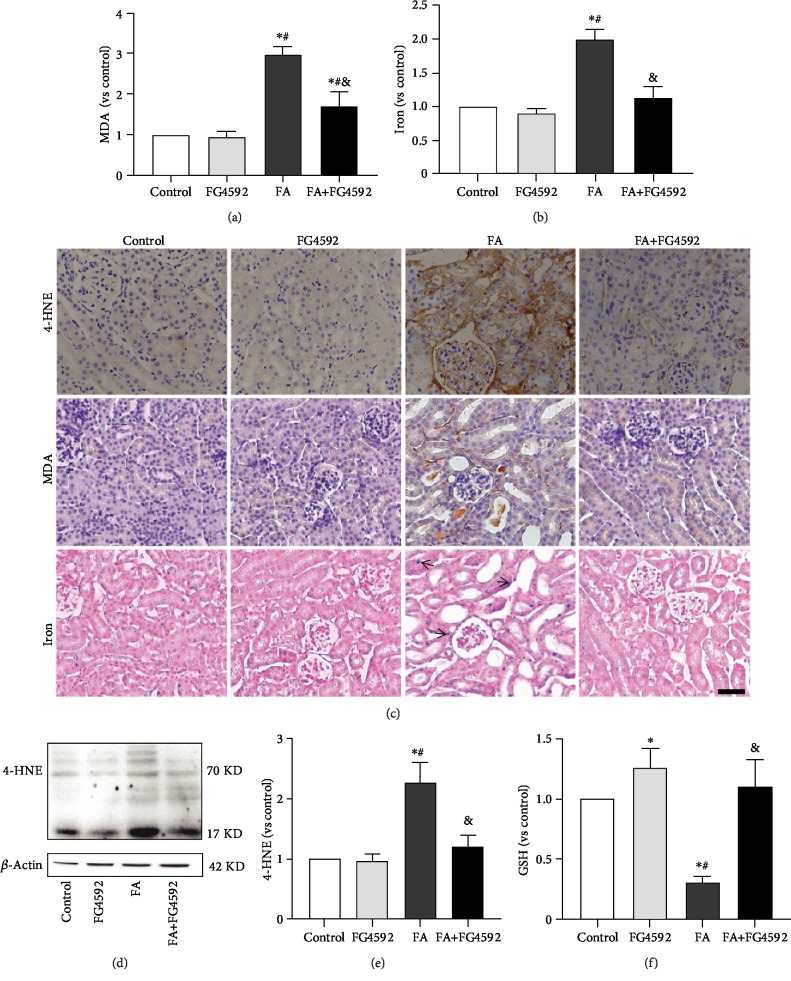
FG-4592 pretreatment alleviates ferroptosis in FA-induced AKI. (a) Renal MDA level in the renal tissues. (b) Iron content. (c) Immunohistochemical staining of 4-HNE and MDA; Perls' staining. Bar, 50 *μ*m. Arrows for iron accumulation. (d) Western blotting of 4-HNE levels in the kidney lysates. (e) Semiquantitative measurements of the 4-HNE levels. (f) GSH concentration. The values are expressed as the means ± SEM. ^∗^*P* < 0.05 versus the control group. ^#^*P* < 0.05 versus the FG-4592 group. ^&^*P* < 0.05 versus the FA group.

To determine if there was ferroptosis-related kidney dysfunction on the second day after FA administration, we detected the levels of lipid peroxidation and iron accumulation. In the FA group, the levels of MDA and iron increased in both tissue and section staining. The expression of 4-HNE also increased in both western blotting and immunohistochemical staining while the GSH tissue level decreased. These changes were reversed in the FA+FG-4592 group, as shown in Figure 2.

To further validate the antiferroptosis effect of FG-4592 pretreatment in FA-induced AKI, the FA-treated mice were administrated with Fer-1. As compared to the FA group, serum levels of BUN and creatinine decreased in the FA+Fer-1 group. Similarly, the histologic analysis showed noticeably alleviated renal injury, immunostaining showed decreased levels of MDA and 4-HNE, and TUNEL staining showed decreased cell death, as shown in Supplementary Fig. [Supplementary-material supplementary-material-1].

### 3.3. FG-4592 Pretreatment Prevented FA-Induced HIF-1*α* Reduction and Enhanced Nrf2 Activation

**Figure 3:**
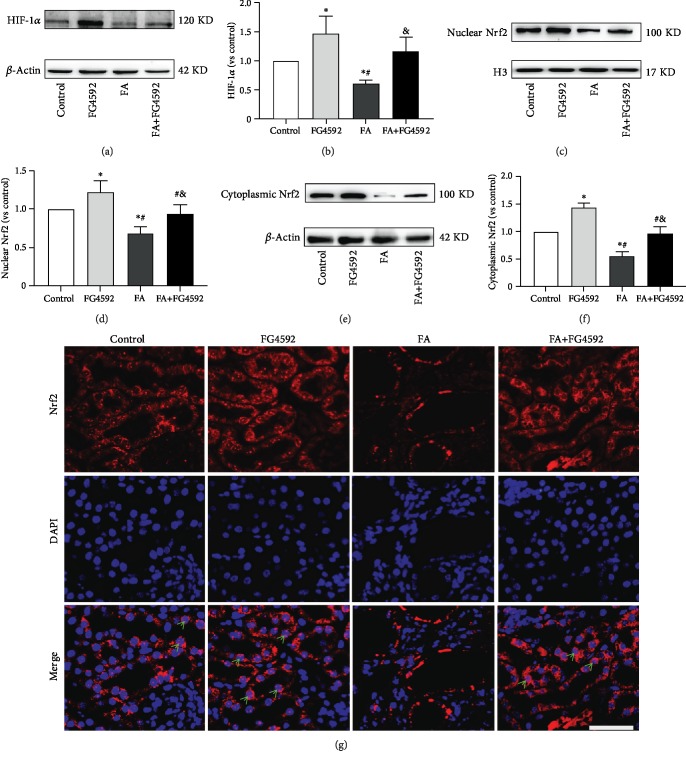
FG-4592 Pretreatment increases the expression of HIF-1*α* and activates Nrf2. (a) Western blotting of the HIF-1*α*. (b) Semiquantitative measurements of HIF-1*α*. (c) Western blotting of nuclear Nrf2. (d) Semiquantitative measurements of nuclear Nrf2. (e) Western blotting of cytoplasmic Nrf2. (f) Semiquantitative measurements of cytoplasmic Nrf2. (g) Immunofluorescence staining showing Nrf2 nuclear translocation (green arrowheads). Bar, 50 *μ*m. The values are expressed as the means ± SEM. ^∗^*P* < 0.05 versus the control group. ^#^*P* < 0.05 versus the FG-4592 group. ^&^*P* < 0.05 versus the FA group.

As shown in Figure 3, we examined the role of FG-4592 pretreatment in HIF-1*α* stabilization and Nrf2 activation using western blotting and immunofluorescence staining. As compared to that in the control group, the expression of HIF-1*α* as well as both cytoplasmic and nuclear Nrf2 significantly decreased in the FA group, while FG-4592 pretreatment reversed the decreased levels. The FG-4592 group showed the highest level among all groups. Likewise, immunofluorescence staining revealed an extensive translocation of Nrf2 in the nuclei in the FG-4592 group and FA+FG-4592 group.

**Figure 4:**
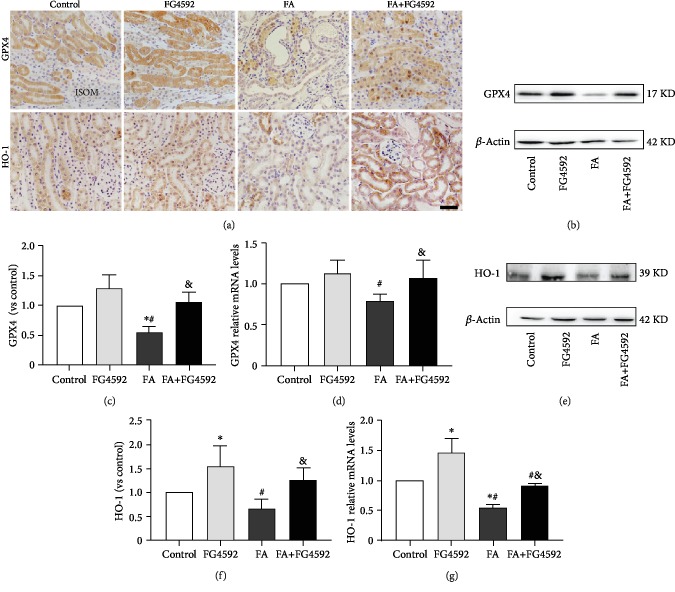
FG-4592 pretreatment upregulates Nrf2-ARE. (a) Immunohistochemical staining of GPX4 and HO-1. ISOM: inner stripe of the outer medulla. Bar, 50 *μ*m. (b) Western blotting of GPX4. (c) Semiquantitative measurements of GPX4. (d) Relative mRNA expression of GPX4. (e) Western blotting of HO-1. (f) Semiquantitative measurements of HO-1. (g) Relative mRNA expression of HO-1. The values are expressed as the means ± SEM. ^∗^*P* < 0.05 versus the control group. ^#^*P* < 0.05 versus the FG-4592 group. ^&^*P* < 0.05 versus the FA group.

**Figure 5:**
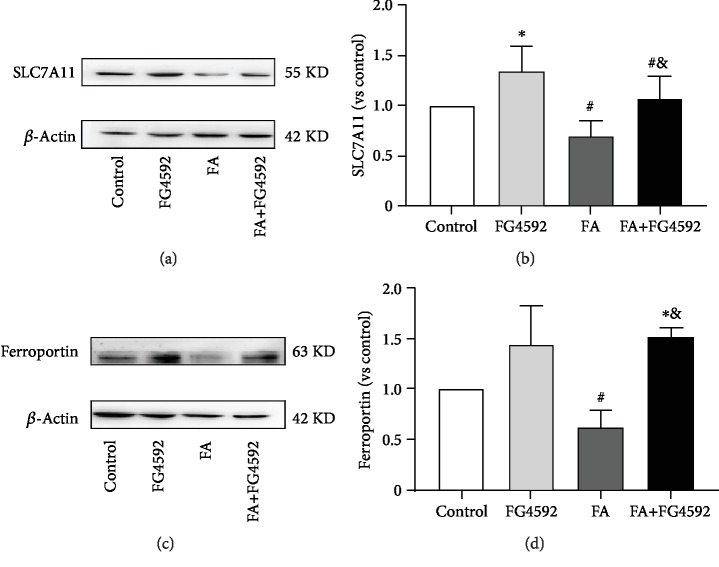
FG-4592 pretreatment increases the expression of Nrf2-mediated SLC7A11 and ferroportin protein expression. (a) Western blotting of SLC7A11. (b) Semiquantitative measurements of SLC7A11. (c) Western blotting of ferroportin. (d) Semiquantitative measurements of ferroportin. The values are expressed as the means ± SEM. ^∗^*P* < 0.05 versus the control group. ^#^*P* < 0.05 versus the FG-4592 group. ^&^*P* < 0.05 versus the FA group.

As shown in Figure 4, immunohistochemical staining demonstrated a lower level of expression of GPX4 and HO-1 in the FA group, compared to that in the control group, while the expression of those in the FA+FG-4592 group increased. Consistently, the FA+FG-4592 group also increased the expression levels of both protein and mRNA of HO-1 and GPX4, two downstream targets of Nrf2, but not as high as that in the FG-4592 group. In addition, two more downstream targets of Nrf2, SLC7A11 and ferroportin, were both upregulated in the FG-4592 group and the FA+FG-4592 group as compared to the FA group (Figure 5), which were consistent with the results of an enhanced GSH level and reduced iron content (Figure 2).

In summary, FG-4592 pretreatment increased the expression of HIF-1*α* and activated the Nrf2 signaling pathway. This implied that FG-4592 pretreatment decreased tubular damage by enhancing the antioxidant capacity and iron turnover, ultimately reducing ferroptosis in the tubular cells.

### 3.4. FG-4592 Pretreatment Decreased Ferroptosis by Akt/GSK-3*β*/Nrf2 Pathway

**Figure 6:**
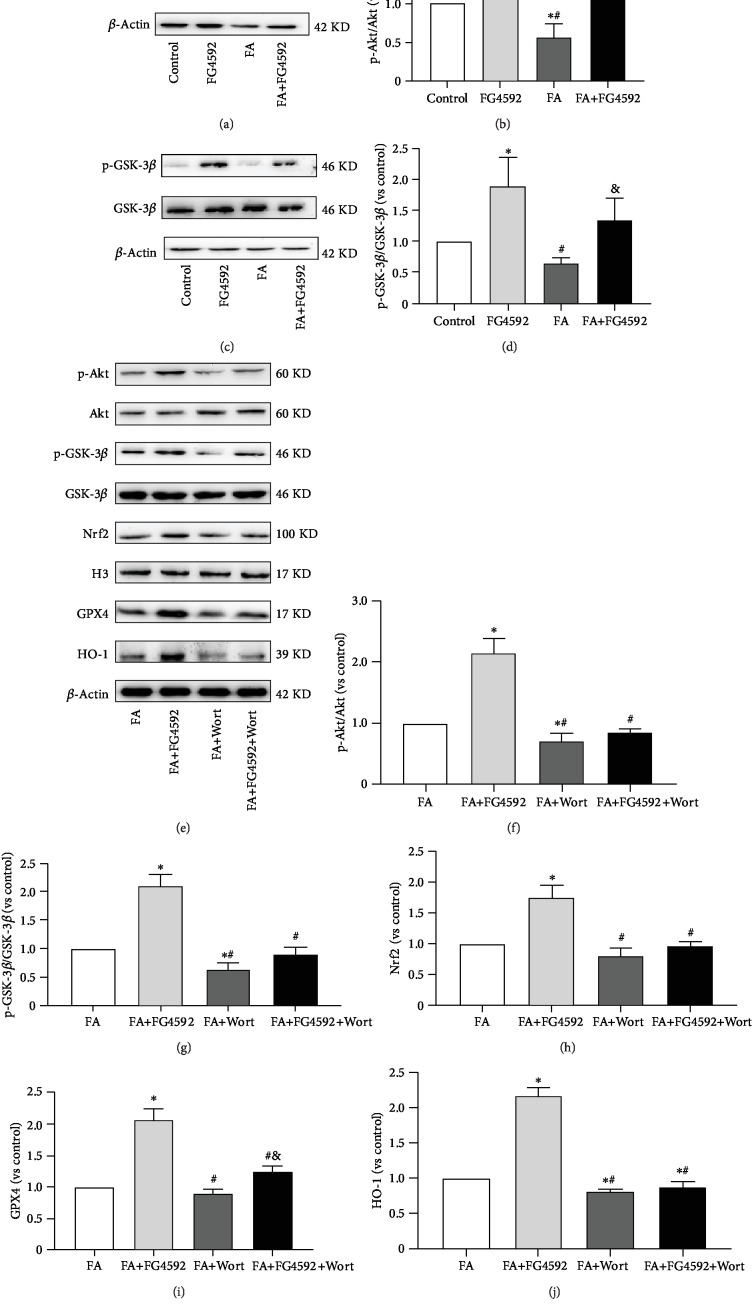
FG-4592 pretreatment increases the phosphorylation of Akt/GSK-3*β* in FA-induced AKI. (a) Western blotting of phosphorylated and total Akt. (b) Semiquantitative measurements of ratio of phosphorylated to total Akt. (c) Western blotting of phosphorylated and total GSK-3*β*. (d) Semiquantitative measurements of ratio of phosphorylated to total GSK-3*β*. The values are expressed as the means ± SEM. ^∗^*P* < 0.05 versus the control group. ^#^*P* < 0.05 versus the FG-4592 group. ^&^*P* < 0.05 versus the FA group; These effects were inhibited by treatment with wortmannin, a PI3K inhibitor. (e) Western blotting of p-Akt and total Akt, p-GSK-3*β* and total GSK-3*β*, nuclear Nrf2, GPX4, and HO-1. Semiquantitative measurements of (f) the ratio of p-Akt to total Akt, (g) ratio of p-GSK-3*β* to total GSK-3*β*, (h) nuclear Nrf2, (i) GPX4, and (j) HO-1. The values are expressed as the means ± SEM. ^∗^*P* < 0.05 versus the FA group. ^#^*P* < 0.05 versus the FA+FG-4592 group. ^&^*P* < 0.05 versus the FA+Wort group.

To explore the mechanism of the upregulation of Nrf2 activity by FG-4592, the level of Akt and GSK-3*β*, upstream proteins of Nrf2, and their phosphorylated levels were examined using western blotting. As shown in Figure 6, the ratios of their phosphorylated levels to their general levels increased in the FG-4592 group and the FA+FG-4592 group as compared to those in the FA group. Meanwhile, the FA group had lower levels of phosphorylated- (p-) Akt and p-GSK-3*β*, as compared to the control group.

To further confirm whether PI3K/Akt was involved in Nrf2 activation, western blotting showed that wortmannin significantly attenuated Akt activation (decreased p-Akt expression) and enhanced GSK-3*β* activation (decreased p-GSK-3*β*), which resulted in the downregulation of nuclear accumulation of Nrf2 and its downstream proteins HO-1 and GPX4. The effect of FG-4592 pretreatment on the activation of Nrf2 was abolished in the presence of the specific inhibitor, as shown in Figure 6.

### 3.5. FG-4592 Pretreatment Decreased Inflammation in FA-Induced AKI

**Figure 7:**
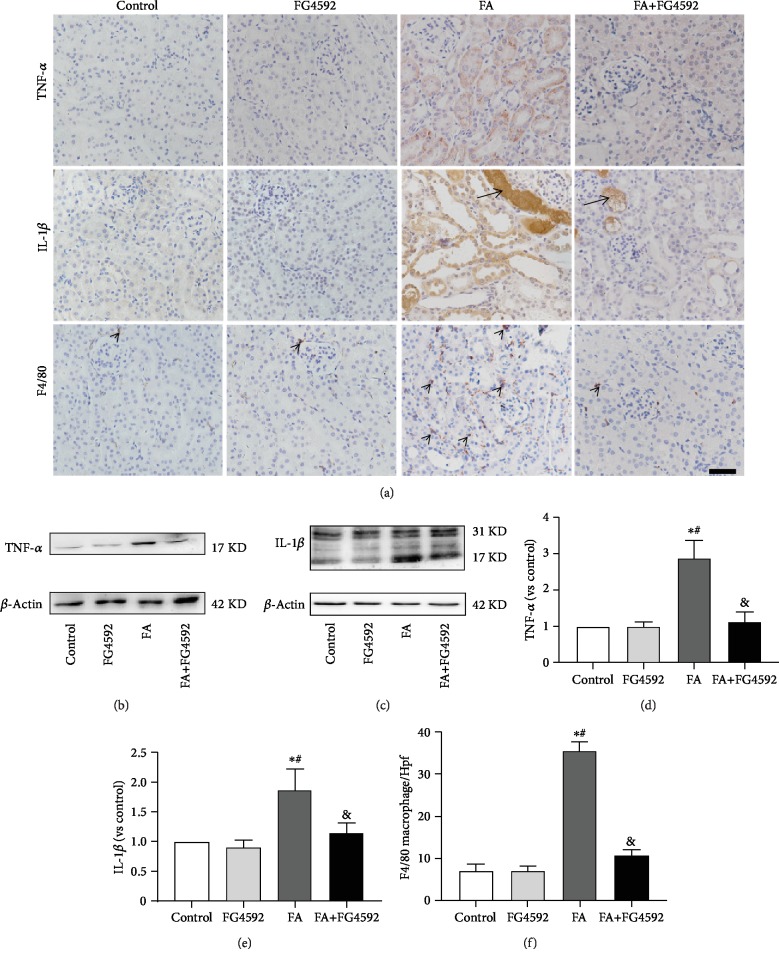
FG-4592 pretreatment decreases expression of TNF*α* and IL-1*β* levels and F4/80 infiltration in FA-induced AKI. (a) Immunohistochemical staining of TNF-*α* (brown), IL-1*β* (brown), and F4/80-positive interstitial macrophages (black arrowheads). Black arrows for folic acid crystals in tubule lumen. Bar, 50 *μ*m. (b) Western blotting of TNF-*α*. (c) Western blotting of IL-1*β*. (d) Semiquantitative measurements of TNF-*α*. (e) Semiquantitative measurements of IL-1*β*. (f) Absolute counting of the number of F4/80-positive interstitial macrophages per high-power field (Hpf). The values are expressed as the means ± SEM. ^∗^*P* < 0.05 versus the control group. ^#^*P* < 0.05 versus the FG-4592 group. ^&^*P* < 0.05 versus the FA group.

Based on an increasing body of evidence, cytokines and macrophages play a pivotal role in the progression of AKI [30]. In the present study, both immunohistochemical staining and western blotting detected lower expression of the proinflammatory cytokines, TNF-*α* and IL-1*β*, in the FA+FG-4592 group as compared to the FA group. Meanwhile, immunohistochemical staining for F4/80, a marker for macrophage, showed significantly decreased expression in the FA+FG-4592 group versus the FA group (Figure 7).

### 3.6. FG-4592 Pretreatment Mitigated FA-Induced Renal Fibrosis on the 14^th^ Day after FA Injection

**Figure 8:**
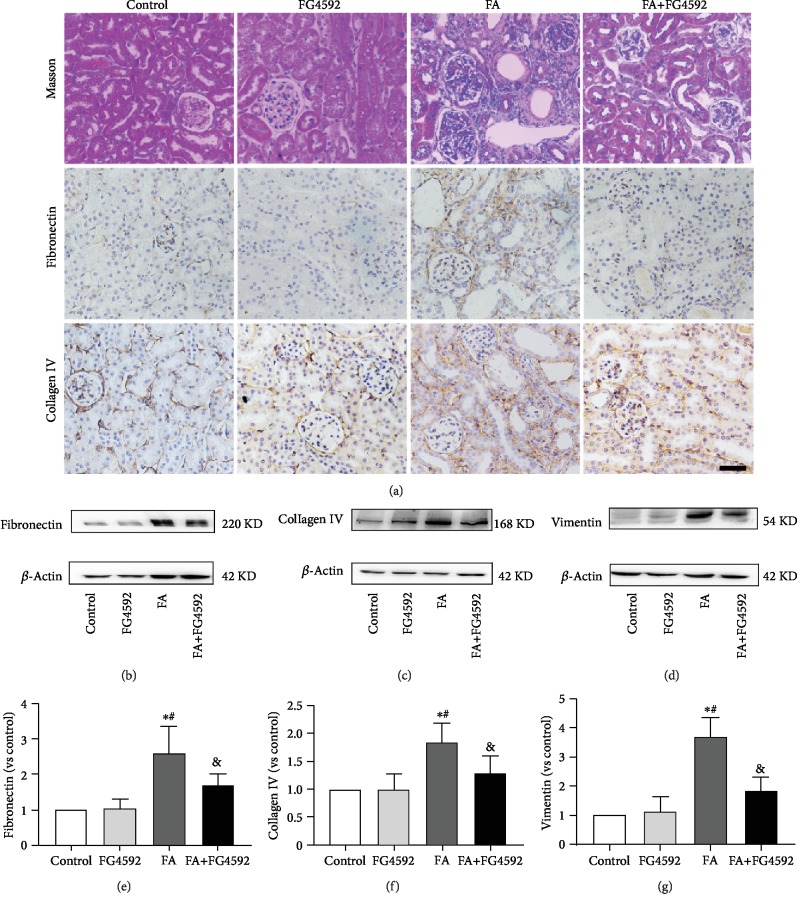
FG-4592 pretreatment restrains fibrosis progression on the 14th day after FA injection. (a) Deposition of fibrils (blue) and infiltration of inflammatory cells around vessels are indicated with Masson's trichrome staining. Bar, 50 *μ*m. Immunostaining of fibronectin and collagen IV is shown in brown. (b) Western blotting of fibronectin. (c) Western blotting of collagen IV. (d) Western blotting of vimentin. (e) Semiquantitative measurement of fibronectin. (f) Semiquantitative measurement of collagen IV. (g) Semiquantitative measurement of vimentin. The values are expressed as the means ± SEM. ^∗^*P* < 0.05 versus the control group. ^#^*P* < 0.05 versus the FG-4592 group. ^&^*P* < 0.05 versus the FA group.

We evaluated the long-term effect of FG-4592 pretreatment on the progression of kidney fibrosis induced by FA injection. To this end, Masson staining showed a decreased collagen deposition in the FA+FG-4592 group as compared to the FA group on the 14^th^ day after FA injection. Immunohistochemical staining of fibronectin and collagen IV exhibited deceased expressions in the FA+FG-4592 group versus the FA group, consistent with the results from western blotting for fibronectin, collagen IV, and vimentin (Figure 8).

## 4. Discussion

Renal tubule damage is a common pathological process of AKI caused by various factors [31]. The transition from AKI to CKD gradually occurs without adequate and prompt treatment [32]. FA-induced kidney injury is characterized by tubule epithelium degeneration, interstitial infiltration of immunocytes, and the release of inflammatory factors in the early stage, as well as an incremental extracellular matrix in the advanced phase [33, 34]. The mechanism of tubular damage has been reported to be associated with a disturbance of the antioxidation system, consequently causing the death of epithelial cells [35–38].

It is well known that exposure to special medical interventions, such as cisplatin for cancer chemotherapy and contrast agent for imaging examination, enhances the risk of AKI. Thus, prophylactic medication before the use of nephrotoxic medicine is promising for protecting the kidney from injury. FG-4592 is an inhibitor of PHD that degrades HIF in normoxia and therefore is a stabilizer of HIF. Currently, FG-4592 is used to improve the anemia of CKD patients since it can enhance the erythropoietin (EPO), a downstream gene of HIF [39]. In the present study, FG-4592 pretreatment was anticipated to protect from FA-induced AKI by stabilizing HIF-1*α* and therefore to strengthen the antioxidation system. According to the analysis of histological and biochemical parameters of kidney tissues and blood samples at multiple time points after FA injection, we selected the second day for representing AKI and the 14^th^ day for representing CKD. As a result, on the second day after FA injection, as compared to the control group, HIF-1*α* expression decreased in the FA group, while FG-4592 pretreatment reversed the decreased levels. It was consistent with the study of Weidemann et al., where HIF transactivation and HIF-1*α* protein are not induced but rather suppressed by cisplatin in renal tubular cells *in vivo and in vitro* [40]. In contrast, another study showed that HIF-1*α* is activated in renal tubules in cisplatin nephropathy [41]. These controversial results may be associated with the HIF biological metabolism, varying with the duration, intensity, and the causes of the injuries, such as ischemia, hypoxia, and nephrotoxic drugs. With FG-4592 pretreatment, HIF-1*α* significantly increased parallel to an alleviated tubule damage and improved renal function indicated by pathological and biochemical parameters. This might be due to the increasing capacity of antioxidative stress. A recent study also showed that HIF-1*α* activation can protect kidneys from CKD development through increasing stress-responsive transcription factors which can be activated for adaptions to counteract hypoxic insults in the renal IRI [42]. In the pilot experiment, we compared the effect of both FG-4592 posttreatment and FG-4592 pretreatment, Masson staining showed more fibrils in the renal interstitial (data not shown) in FG-4592 posttreatment. This result is consistent with the finding that HIF activation in the UUO model aggravates fibrosis [43]. Another study has also shown that preischemic targeting of HIF prolyl hydroxylation inhibits fibrosis caused by renal IRI, while postischemic PHD inhibition does not impact AKI-associated fibrosis [44]. These data suggest that HIF-1 dependent transcriptional activation appeared to change with a time-dependent manner, which affected the progression of fibrosis.

To explore the protective mechanism of FG-4592 pretreatment, we detected one of the HIF-1*α* regulating proteins, Nrf2, a transcriptive factor, as well as downstream genes of Nrf2. It was reported that HIF-1*α* can activate the Nrf2-ARE pathway protecting organs from injuries in some diseases [24, 26, 27]. In this study, the upregulation of Nrf2 was observed, and the expressions of its downstream proteins were also correspondingly upregulated, including GPX4, HO-1, SLC7A11, and ferroportin. According to the common hypothesis, GPX4 reduces ferroptosis in the presence of GSH with SLC7A11 mediation [20, 45, 46]. The recent study has shown that circadian control of brain-derived neurotrophic factor- (BDNF-) mediated Nrf2 activation protects dopaminergic neurons from ferroptosis by increasing GSH synthesis [47]. It has been reported that HO-1 has the capacity to catalyze the breakdown of the prooxidant heme into biliverdin, acting as an antioxidant against ferroptosis [48, 49]. In addition, HO-1 is observed to upregulate ferritin; therefore, another role of HO-1 is to regulate iron metabolism [50], together with ferroportin [18, 51, 52]. Studies have shown that glycyrrhizin activates the Nrf2-ARE pathway and protects liver cell against ferroptosis through upregulation of antioxidant enzymes, e.g., GPX4 and HO-1 [53]. Moreover, ferroportin activated by the Nrf2 improves iron mobility and therefore is involved in antiferroptosis [54]. Several studies also show that recovery of the ferroportin expression could alleviate ferroptosis in cardiomyocytes and neurons [52, 55]. Lipid peroxidation, decreased by increased downstream antioxidative enzymes of Nrf2, and iron accumulation, decreased by increased ferroportin, are the main causes for cellular ferroptosis [56]. Our results were consistent with these mechanisms, i.e., lipid peroxidation and iron accumulation decreased as GSH, and antioxidative enzymes and ferroportin increased with the FG-4592 pretreatment. Thus, the protective effect of the FG-4592 pretreatment may be associated with antiferroptosis via Nrf2 activation. To verify this, Fer-1, the first generation of ferroptosis inhibitor [57], was administrated in FA-treated mice. As a result, the antiferroptosis effect of Fer-1 was demonstrated by improved renal function and pathological parameters, which confirmed the hypothesis that ferroptosis might be the principal pathological process in FA-induced AKI.

In addition, as a mechanism underlying the activation of Nrf2, Keap1 is accepted to be the main governor of Nrf2 activity. In a state of low oxidative stress, the cysteine residues on Keap1 are oxidized, leading to the release of Nrf2 from the Keap1/Nrf2 complex and subsequent translocation into the nucleus [29]. However, in the present study, there was no significant difference in the expression of Keap1 between the control group and FG-4592 group, as well as between the FA group and FA+FG-4592 group (as shown in Supplementary Fig. [Supplementary-material supplementary-material-1]), suggesting FG-4592 mediated changes in Nrf2 expression may not be due to the regulation of Keap1 expression level. It has been reported that GSK-3*β*-mediated Keap1-independent regulatory pathway plays a key role in both AKI and CKD caused by severe oxidative stress injury [58, 59]. What is more, impaired activation of Nrf2 is involved in severe oxidative stress injury [60]. In our study, the transcriptive activity of Nrf2 in the FA group was demonstrated to be diminished, and FG-4592 pretreatment reversed the level of Nrf2. It is speculated that the GSK-3*β* mediated Keap1-independent regulation may occur mainly with FG-4592 pretreatment. It has been reported that salvianolic acid A prevents progression of CKD by activating the Akt/GSK-3*β*/Nrf2 signaling pathway in 5/6 nephrectomized rats [23]. Another study also suggests that fenofibrate prevents the progress of diabetic nephropathy via activating the PI3K/Akt/GSK-3*β*/Nrf2 pathway [58]. Our results indicated that p-Akt and p-GSK-3*β* decreased in the FA group, while they were upregulated with FG-4592 pretreatment. It was consistent with the fact that HIF-1*α* is involved in the activation of the Akt signaling pathways [61, 62] and the phosphorylated Akt/GSK-3*β* prevents the nuclear export of Nrf2, thus maintaining Nrf2 transcriptional function in the nuclei [63]. To further clarify the hypothesis that activation of the PI3K/Akt pathway played a crucial role in modulating Nrf2 expression, we showed that Nrf2 activation was abolished by treatment with a PI3K inhibitor through the inhibition of Akt and GSK-3*β* phosphorylation.

What is more, ferroptosis and inflammation were inseparably linked as they generated a vicious cycle. FG-4592 pretreatment played a role in anti-inflammation on the second day after FA injection. It led to the downregulation of inflammatory factors TNF-*α* and IL-1*β* and the decreased expression of F4/80 (a macrophage marker). Ferroptosis is a regulated cell death, which is more immunogenic and proinflammatory. The study has shown that ferroptosis triggers initial inflammation in steatohepatitis and may be a therapeutic target for preventing the onset of steatohepatitis [64]. Ferroptosis inhibition may result in the prohibition of the inflammatory cascade in the spinal cord injury [65]. Our results suggested that ferroptosis may be a driver of inflammatory responses; however, the upregulation of cytokines and macrophage infiltration could be both quenched by FG-4592 pretreatment.

Furthermore, ferroptosis and inflammation have been reported to be implicated in the development of organ fibrosis, although the mechanism remains uncertain [66–68]. The recent study suggests that ferroptosis plays a critical role in radiation-induced lung fibrosis (RILF) and ferroptosis inhibitor liproxstatin-1 alleviates RILF by decreasing ferroptosis and inflammation via Nrf2-mediated upregulation of HO-1 and GPX4 [67]. One of the driving factors in progressive interstitial fibrosis after AKI could be interstitial inflammation. Inflammation is involved in the pathogenesis of AKI and plays a key role in the following transition to CKD [34]. The present study revealed that FG-4592 pretreatment decreased the expression of collagen IV, fibronectin, and vimentin, the parameters representing the level of renal fibrosis. Hence, the results above may imply that the alleviation of renal fibrosis was associated with the decreased ferroptosis and inflammation at the early stage of kidney injury.

In conclusion, FG-4592 pretreatment plays a protective role at the early stage of FA-induced renal injury through alleviating ferroptosis, which may be achieved by stabilizing HIF-1*α* and activating the Nrf2 signaling pathway. Accordingly, alleviating ferroptosis and subsequently suppressing inflammation could have a long-term protective effect against fibrosis. Thus, the present study emphasized the use of FG-4592 pretreatment as a preventive approach to improve the prognosis of AKI in the clinic. Hopefully, the complex protective roles of FG-4592 would be further disclosed in the near future, such as whether more signal pathways are involved in the regulation of the antioxidative capacity.

## Data Availability

The [DATA TYPE] data used to support the findings of this study are included within the article.

## Supplementary Materials

Supplementary Materials Supplemental Fig. 1: the renoprotective effects of Fer-1 treatment in FA-induced AKI. (A and B) Renal function is assessed by (A) plasma creatinine and (B) BUN levels. (C) Renal tubular injury was assessed by H&E staining; immunohistochemical staining of 4-HNE and MDA Bar, 50 *μ*m. (D) Kidney tubular injury score based on H&E staining. (E) Quantification of 4-HNE. (F) Quantification of MDA. The values are expressed as the means ± SEM. (*n* = 6) ^∗^*P* < 0.05 versus the control group. ^#^*P* < 0.05 versus the FA group. Supplemental Fig. 2: effects of FG-4592 pretreatment on the Keap1/Nrf2 pathway in FA-induced AKI. (A) Western blotting of Keap1. (B) Semiquantitative measurements of Keap1. The values are expressed as the means ± SEM. ^∗^*P* < 0.05versus the control group. ^#^*P* < 0.05 versus the FG-4592 group. ^&^*P* < 0.05 versus the FA group.

## References

[1] Siew E. D., Parr S. K., Abdel-Kader K. (2016). Predictors of recurrent AKI. *Journal of the American Society of Nephrology*.

[2] Patel S. S., Palant C. E., Mahajan V., Chawla L. S. (2017). Sequelae of AKI. *Best Practice & Research Clinical Anaesthesiology*.

[3] Chawla L. S., Kimmel P. L. (2012). Acute kidney injury and chronic kidney disease: an integrated clinical syndrome. *Kidney International*.

[4] Ishani A., Xue J. L., Himmelfarb J. (2009). Acute kidney injury increases risk of ESRD among elderly. *Journal of the American Society of Nephrology*.

[5] Doi K., Rabb H. (2016). Impact of acute kidney injury on distant organ function: recent findings and potential therapeutic targets. *Kidney International*.

[6] Stallons L. J., Whitaker R. M., Schnellmann R. G. (2014). Suppressed mitochondrial biogenesis in folic acid-induced acute kidney injury and early fibrosis. *Toxicology Letters*.

[7] Aparicio-Trejo O. E., Reyes-Fermin L. M., Briones-Herrera A. (2019). Protective effects of N-acetyl-cysteine in mitochondria bioenergetics, oxidative stress, dynamics and S-glutathionylation alterations in acute kidney damage induced by folic acid. *Free Radical Biology and Medicine*.

[8] Metz-Kurschel U., Kurschel E., Wagner K., Aulbert E., Graben N., Philipp T. (1990). Folate nephropathy occurring during cytotoxic chemotherapy with high-dose folinic acid and 5-fluorouracil. *Renal Failure*.

[9] Ostermann M., Liu K. (2017). Pathophysiology of AKI. *Best Practice & Research Clinical Anaesthesiology*.

[10] Gupta A., Puri V., Sharma R., Puri S. (2012). Folic acid induces acute renal failure (ARF) by enhancing renal prooxidant state. *Experimental and Toxicologic Pathology*.

[11] Justo P., Sanz A. B., Sanchez-Nino M. D. (2006). Cytokine cooperation in renal tubular cell injury: the role of TWEAK. *Kidney International*.

[12] Guerrero-Hue M., Garcia-Caballero C., Palomino-Antolin A. (2019). Curcumin reduces renal damage associated with rhabdomyolysis by decreasing ferroptosis-mediated cell death. *The FASEB Journal*.

[13] Linkermann A., Chen G., Dong G., Kunzendorf U., Krautwald S., Dong Z. (2014). Regulated cell death in AKI. *Journal of the American Society of Nephrology*.

[14] Dixon S. J., Lemberg K. M., Lamprecht M. R. (2012). Ferroptosis: an iron-dependent form of nonapoptotic cell death. *Cell*.

[15] Li Q., Li Q. Q., Jia J. N. (2019). Baicalein exerts neuroprotective effects in FeCl3-induced posttraumatic epileptic seizures *via* suppressing ferroptosis. *Frontiers in Pharmacology*.

[16] Martin-Sanchez D., Ruiz-Andres O., Poveda J. (2017). Ferroptosis, but not necroptosis, is important in nephrotoxic folic acid–induced AKI. *Journal of the American Society of Nephrology*.

[17] Dodson M., Castro-Portuguez R., Zhang D. D. (2019). NRF2 plays a critical role in mitigating lipid peroxidation and ferroptosis. *Redox Biology*.

[18] Kerins M. J., Ooi A. (2018). The roles of NRF2 in modulating cellular iron homeostasis. *Antioxidants & Redox Signaling*.

[19] Abdalkader M., Lampinen R., Kanninen K. M., Malm T. M., Liddell J. R. (2018). Targeting Nrf2 to suppress ferroptosis and mitochondrial dysfunction in neurodegeneration. *Frontiers in Neuroscience*.

[20] Yang W. S., SriRamaratnam R., Welsch M. E. (2014). Regulation of ferroptotic cancer cell death by GPX4. *Cell*.

[21] Gao X., Guo N., Xu H. (2019). Ibuprofen induces ferroptosis of glioblastoma cells via downregulation of nuclear factor erythroid 2-related factor 2 signaling pathway. *Anti-Cancer Drugs*.

[22] Nezu M., Souma T., Yu L. (2017). Transcription factor Nrf2 hyperactivation in early-phase renal ischemia-reperfusion injury prevents tubular damage progression. *Kidney International*.

[23] Nezu M., Suzuki N., Yamamoto M. (2017). Targeting the KEAP1-NRF2 system to prevent kidney disease progression. *American Journal of Nephrology*.

[24] Zhang H. F., Wang J. H., Wang Y. L. (2019). Salvianolic acid a protects the kidney against oxidative stress by activating the Akt/GSK-3*β*/Nrf2 signaling pathway and inhibiting the NF-*κ*B signaling pathway in 5/6 nephrectomized rats. *Oxidative Medicine and Cellular Longevity*.

[25] Jiang L., Zeng H., Ni L. (2019). HIF-1*α* preconditioning potentiates antioxidant activity in ischemic injury: the role of sequential administration of dihydrotanshinone I and protocatechuic aldehyde in cardioprotection. *Antioxidants & Redox Signaling*.

[26] Smythies J. A., Sun M., Masson N. (2019). InherentDNA‐binding specificities of theHIF-1*α* andHIF-2*α* transcription factors in chromatin. *EMBO Reports*.

[27] Wu K., Zhou K., Wang Y. (2016). Stabilization of HIF-1*α* by FG-4592 promotes functional recovery and neural protection in experimental spinal cord injury. *Brain Research*.

[28] Ji W., Wang L., He S. (2018). Effects of acute hypoxia exposure with different durations on activation of Nrf2-ARE pathway in mouse skeletal muscle. *PLoS One*.

[29] Maxwell P. H., Eckardt K. U. (2016). HIF prolyl hydroxylase inhibitors for the treatment of renal anaemia and beyond. *Nature Reviews. Nephrology*.

[30] Linkermann A., Stockwell B. R., Krautwald S., Anders H. J. (2014). Regulated cell death and inflammation: an auto-amplification loop causes organ failure. *Nature Reviews. Immunology*.

[31] Agarwal A., Dong Z., Harris R. (2016). Cellular and molecular mechanisms of AKI. *Journal of the American Society of Nephrology*.

[32] Kaushal G. P., Shah S. V. (2014). Challenges and advances in the treatment of AKI. *Journal of the American Society of Nephrology*.

[33] Doi K., Okamoto K., Negishi K. (2006). Attenuation of folic acid-induced renal inflammatory injury in platelet-activating factor receptor-deficient mice. *The American Journal of Pathology*.

[34] Jiang C., Zhu W., Yan X. (2016). Rescue therapy with Tanshinone IIA hinders transition of acute kidney injury to chronic kidney disease *via* targeting GSK3*β*. *Scientific Reports*.

[35] Gu Y., Huang F., Wang Y. (2018). Connexin32 plays a crucial role in ROS-mediated endoplasmic reticulum stress apoptosis signaling pathway in ischemia reperfusion-induced acute kidney injury. *Journal of Translational Medicine*.

[36] Wang Z., Huang W., Li H. (2018). Synergistic action of inflammation and lipid dysmetabolism on kidney damage in rats. *Renal Failure*.

[37] Meng X. M., Ren G. L., Gao L. (2018). NADPH oxidase 4 promotes cisplatin-induced acute kidney injury via ROS-mediated programmed cell death and inflammation. *Laboratory Investigation*.

[38] Skouta R., Dixon S. J., Wang J. (2014). Ferrostatins inhibit oxidative lipid damage and cell death in diverse disease models. *Journal of the American Chemical Society*.

[39] Provenzano R., Besarab A., Sun C. H. (2016). Oral hypoxia–inducible factor prolyl hydroxylase inhibitor roxadustat (FG-4592) for the treatment of anemia in patients with CKD. *Clinical Journal of the American Society of Nephrology*.

[40] Weidemann A., Bernhardt W. M., Klanke B. (2008). HIF activation protects from acute kidney injury. *Journal of the American Society of Nephrology*.

[41] Yang Y., Yu X., Zhang Y. (2018). Hypoxia-inducible factor prolyl hydroxylase inhibitor roxadustat (FG-4592) protects against cisplatin-induced acute kidney injury. *Clinical Science*.

[42] Li L., Kang H., Zhang Q., D'Agati V. D., Al-Awqati Q., Lin F. (2019). FoxO3 activation in hypoxic tubules prevents chronic kidney disease. *The Journal of Clinical Investigation*.

[43] Higgins D. F., Kimura K., Bernhardt W. M. (2007). Hypoxia promotes fibrogenesis in vivo via HIF-1 stimulation of epithelial-to-mesenchymal transition. *The Journal of Clinical Investigation*.

[44] Kapitsinou P. P., Jaffe J., Michael M. (2012). Preischemic targeting of HIF prolyl hydroxylation inhibits fibrosis associated with acute kidney injury. *American Journal of Physiology Renal Physiology*.

[45] Friedmann Angeli J. P., Schneider M., Proneth B. (2014). Inactivation of the ferroptosis regulator Gpx4 triggers acute renal failure in mice. *Nature Cell Biology*.

[46] Sun Y., Zheng Y., Wang C., Liu Y. (2018). Glutathione depletion induces ferroptosis, autophagy, and premature cell senescence in retinal pigment epithelial cells. *Cell Death & Disease*.

[47] Ishii T., Warabi E., Mann G. E. (2019). Circadian control of BDNF-mediated Nrf2 activation in astrocytes protects dopaminergic neurons from ferroptosis. *Free Radical Biology and Medicine*.

[48] Kasai S., Mimura J., Ozaki T., Itoh K. (2018). Emerging regulatory role of Nrf2 in iron, heme, and hemoglobin metabolism in physiology and disease. *Frontiers in Veterinary Science*.

[49] Chen D. Q., Feng Y. L., Chen L. (2019). Poricoic acid A enhances melatonin inhibition of AKI-to-CKD transition by regulating Gas6/AxlNF*κ*B/Nrf2 axis. *Free Radical Biology and Medicine*.

[50] Adedoyin O., Boddu R., Traylor A. (2018). Heme oxygenase-1 mitigates ferroptosis in renal proximal tubule cells. *American Journal of Physiology Renal Physiology*.

[51] Geng N., Shi B. J., Li S. L. (2018). Knockdown of ferroportin accelerates erastin-induced ferroptosis in neuroblastoma cells. *European Review for Medical and Pharmacological Sciences*.

[52] Hou L., Huang R., Sun F., Zhang L., Wang Q. (2019). NADPH oxidase regulates paraquat and maneb-induced dopaminergic neurodegeneration through ferroptosis. *Toxicology*.

[53] Wang Y., Chen Q., Shi C., Jiao F., Gong Z. (2019). Mechanism of glycyrrhizin on ferroptosis during acute liver failure by inhibiting oxidative stress. *Molecular Medicine Reports*.

[54] Kajarabille N., Latunde-Dada G. O. (2019). Programmed cell-death by ferroptosis: antioxidants as mitigators. *International Journal of Molecular Sciences*.

[55] Baba Y., Higa J. K., Shimada B. K. (2018). Protective effects of the mechanistic target of rapamycin against excess iron and ferroptosis in cardiomyocytes. *American Journal of Physiology Heart and Circulatory Physiology*.

[56] Xie Y., Hou W., Song X. (2016). Ferroptosis: process and function. *Cell Death & Differentiation*.

[57] Zilka O., Shah R., Li B. (2017). On the mechanism of cytoprotection by ferrostatin-1 and liproxstatin-1 and the role of lipid peroxidation in ferroptotic cell death. *ACS Central Science*.

[58] Cheng Y., Zhang J., Guo W. (2016). Up-regulation of Nrf2 is involved in FGF21-mediated fenofibrate protection against type 1 diabetic nephropathy. *Free Radical Biology and Medicine*.

[59] Tongqiang L., Shaopeng L., Xiaofang Y. (2016). Salvianolic acid B prevents iodinated contrast media-induced acute renal injury in rats via the PI3K/Akt/Nrf2 pathway. *Oxidative Medicine and Cellular Longevity*.

[60] Wu J., Liu X., Fan J. (2014). Bardoxolone methyl (BARD) ameliorates aristolochic acid (AA)-induced acute kidney injury through Nrf2 pathway. *Toxicology*.

[61] Nouri F., Nematollahi-Mahani S. N., Sharifi A. M. (2019). Preconditioning of mesenchymal stem cells with non-toxic concentration of hydrogen peroxide against oxidative stress induced cell death: the role of hypoxia-inducible factor-1. *Advanced Pharmaceutical Bulletin*.

[62] Nouri F., Salehinejad P., Nematollahi-Mahani S. N., Kamarul T., Zarrindast M. R., Sharifi A. M. (2016). Deferoxamine preconditioning of neural-like cells derived from human Wharton's jelly mesenchymal stem cells as a strategy to promote their tolerance and therapeutic potential: an in vitro study. *Cellular and Molecular Neurobiology*.

[63] Xin Y., Bai Y., Jiang X. (2018). Sulforaphane prevents angiotensin II-induced cardiomyopathy by activation of Nrf2 via stimulating the Akt/GSK-3ss/Fyn pathway. *Redox Biology*.

[64] Tsurusaki S., Tsuchiya Y., Koumura T. (2019). Hepatic ferroptosis plays an important role as the trigger for initiating inflammation in nonalcoholic steatohepatitis. *Cell Death & Disease*.

[65] Zhang Y., Sun C., Zhao C. (2019). Ferroptosis inhibitor SRS 16-86 attenuates ferroptosis and promotes functional recovery in contusion spinal cord injury. *Brain Research*.

[66] Guo J., Guan Q., Liu X. (2016). Relationship of clusterin with renal inflammation and fibrosis after the recovery phase of ischemia-reperfusion injury. *BMC Nephrology*.

[67] Li X., Duan L., Yuan S., Zhuang X., Qiao T., He J. (2019). Ferroptosis inhibitor alleviates radiation-induced lung fibrosis (RILF) via down-regulation of TGF-*β*1. *Journal of Inflammation*.

[68] Chen J., Li D. (2018). Telbivudine attenuates UUO-induced renal fibrosis via TGF-*β*/Smad and NF-*κ*B signaling. *International Immunopharmacology*.

