# Supplementary material for: Pretreatment with Roxadustat (FG-4592) Attenuates Folic Acid-Induced Kidney Injury through Antiferroptosis via Akt/GSK-3β/Nrf2 Pathway
Source: Oxid Med Cell Longev. 2020 Jan 17;2020:6286984. doi: 10.1155/2020/6286984 (PMC6995323; doi:10.1155/2020/6286984)
Supplement: Supplementary Materials — Supplemental Fig. 1: the renoprotective effects of Fer-1 treatment in FA-induced AKI. (A and B) Renal function is assessed by (A) plasma creatinine and (B) BUN levels. (C) Renal tubular injury was assessed by H&E staining; immunohistochemical staining of 4-HNE and MDA Bar, 50 μm. (D) Kidney tubular injury score based on H&E staining. (E) Quantification of 4-HNE. (F) Quantification of MDA. The values are expressed as the means ± SEM. (n = 6) ∗P < 0.05 versus the control group. #P < 0.05 versus the FA group. Supplemental Fig. 2: effects of FG-4592 pretreatment on the Keap1/Nrf2 pathway in FA-induced AKI. (A) Western blotting of Keap1. (B) Semiquantitative measurements of Keap1. The values are expressed as the means ± SEM. ∗P < 0.05versus the control group. #P < 0.05 versus the FG-4592 group. &P < 0.05 versus the FA group. [file 6286984.f1.pdf]

Fig. S1

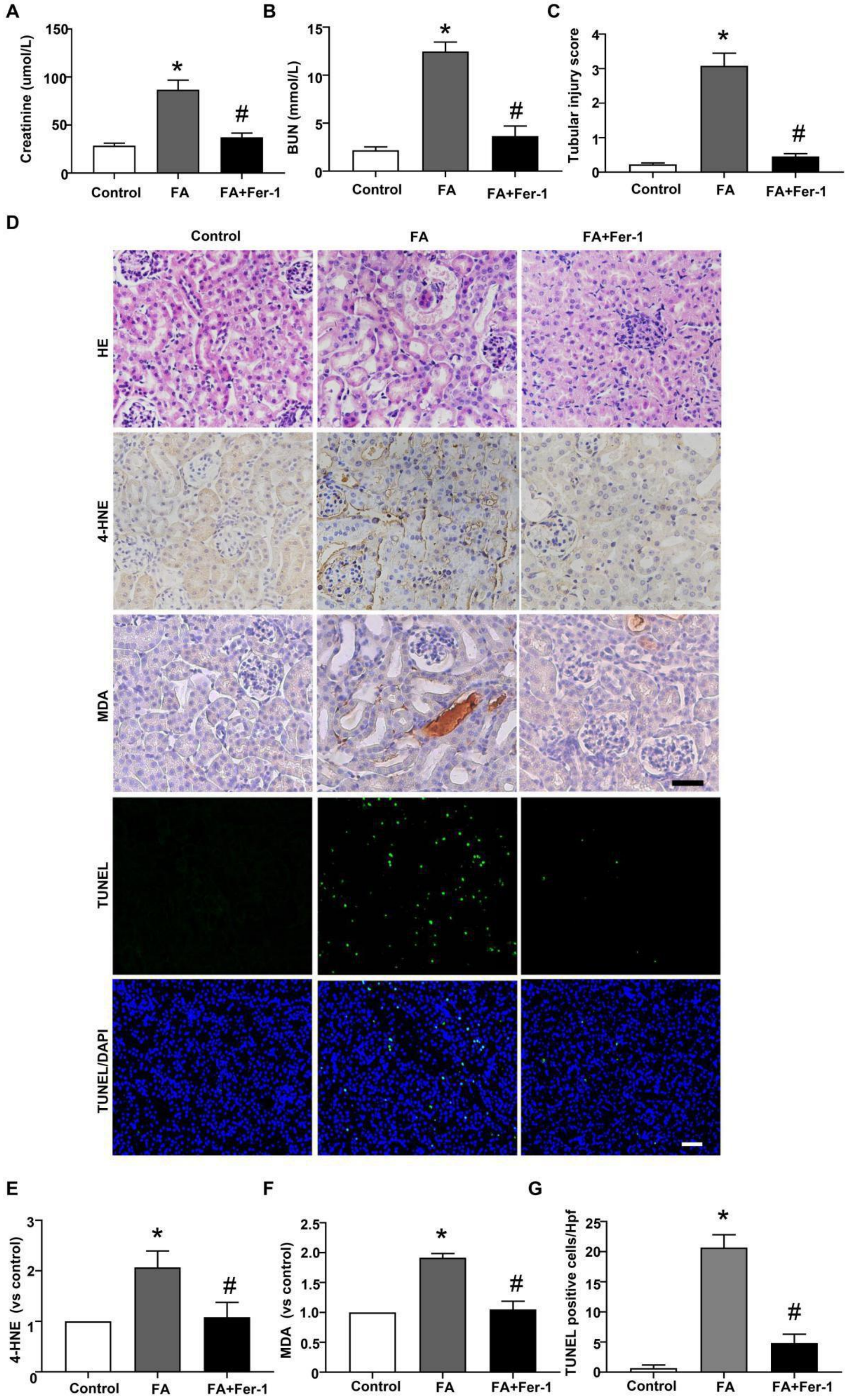

A
Creatinine (umol/L)
Control
FA
FA+Fer-1
B
BUN (mmol/L)
C
Tubular injury score
D
HE
4-HNE
MDA
TUNEL
TUNEL/DAPI
E
4-HNE (vs control)
F
MDA (vs control)
G
TUNEL positive cells/Hpf

Supplemental Fig. 1. The renoprotective effects of Fer-1 treatment in FA-AKI. (A and B) Renal function is assessed by (A) plasma creatinine and (B) BUN levels. (C) Kidney tubular injury score based on H&E staining. (D) Renal tubular injury was assessed by H&E staining; immunohistochemical staining of 4-HNE and MDA; cell death was assessed by TUNEL staining (green). Bar, 50 μm. (E) Quantification of 4-HNE. (F) Quantification of MDA. (G) Absolute counting of the number of TUNEL-positive nuclei per high-power field (Hpf). The values are expressed as the means ± SEM. $^{*}P<0.05$ versus the control group. $^{\#}P<0.05$ versus the FA group.

Fig. S2

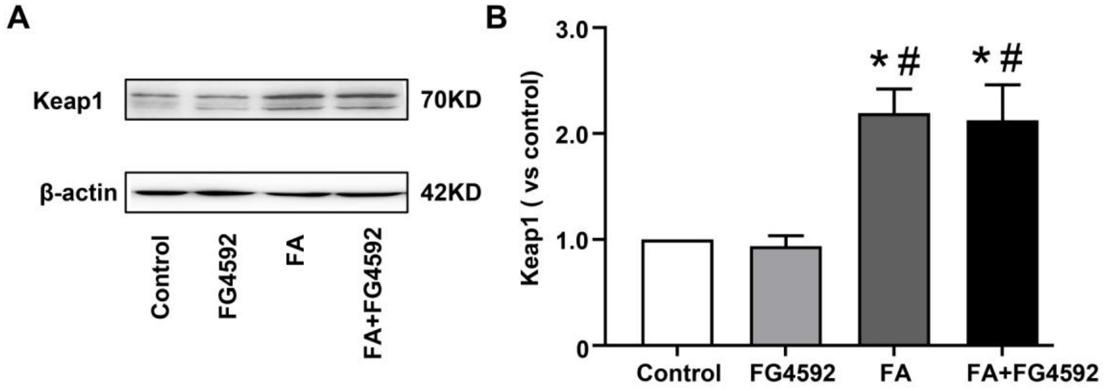

Supplemental Fig. 2. Effects of FG-4592 pretreatment on Keap1/Nrf2 pathway in FA-induced AKI. (A) Western blotting of Keap1. (B) Semi-quantitative measurements of Keap1. The values are expressed as the means ± SEM. $^{*}P$<0.05 versus the control group. $^{\#}P$<0.05 versus the FG-4592 group. $^{\&}P$<0.05 versus the FA group.
